# Wireless Inertial Measurement Units in Performing Arts

**DOI:** 10.3390/s25196188

**Published:** 2025-10-06

**Authors:** Emmanuel Fléty, Frédéric Bevilacqua

**Affiliations:** UMR 9912, STMS Lab IRCAM-CNRS-Sorbonne Université, 75004 Paris, France; emmanuel.flety@ircam.fr

**Keywords:** IMU, wireless, gestural interaction, computer music, performing arts

## Abstract

Inertial Measurement Units (IMUs), which embed several sensors (accelerometers, gyroscopes, magnetometers) are employed by musicians and performers to control sound, music, or lighting on stage. In particular, wireless IMU systems in the performing arts require particular attention due to strict requirements regarding streaming sample rate, latency, power consumption, and programmability. This article presents a review of systems developed in this context at IRCAM as well as in other laboratories and companies, highlighting specificities in terms of sensing, communication, performance, digital processing, and usage. Although basic IMUs are now widely integrated into IoT systems and smartphones, the availability of complete commercial wireless systems that meet the constraints of the performing arts remains limited. For this reason, a review of systems used in performing Arts provides exemplary use cases that may also be relevant to other applications.

## 1. Introduction

The invention of the Theremin in the early 20th century [1] was one of the first musical systems to enable gestures to control sound synthesis. Taking then advantage of the invention of vacuum tubes, it marked the beginning of the development of musical instruments using electrical/electronic technologies. Since then, this concept has been the subject of sustained research, and a wide range of devices, from experimental prototypes to commercial products, have been proposed and adopted by artists. At the turn of the 21st century, stimulated by the increased availability of motion-sensing devices and music software, a large interdisciplinary community of musicians, designers, and engineers emerged around the international conference series “New Interfaces for Musical Expression” (https://nime.org, accessed on 17 May 2025). Related research has also been presented at conferences in Human-Computer Interaction (e.g., ACM CHI, TEI, DIS), Movement and Computing (MoCo), and other music technology conferences (e.g., Sound and Music Computing, International Computer Music Conference).

Over the years, these communities have reported on the evolution of wireless motion sensing and real-time processing, as well as their application in artistic projects. As we will describe, these research and artistic communities face specific constraints and requirements, both technical—such as latency and interoperability between hardware and software—and economic. In this paper, we focus specifically on the use of wireless Inertial Measurement Units (IMU) in the performing arts. For these reasons, this review does not address the general use of IMUs in IoT, but rather their artistic applications. As first highlighted by Perry Cook in 2001 [2], the use of wireless motion sensing poses challenges for artistic performances. These real-time technologies are inherently difficult to implement while maintaining sufficient bandwidth in environments with radio-frequency interference. Interestingly, the requirements for artistic performance remain distinct from those of standard wireless communication and IoT applications, particularly due to the need for low-latency real-time interaction. Furthermore, given constraints of cost and availability, only low-cost, widely available IMUs are typically used in the performing arts, which imposes limitations on possible data processing. In addition, movement analysis in artistic applications requires specific approaches, adaptable to wide range of user expertise. For these reasons, we believe that a review of different devices and data processing methods is particularly valuable for a broad community of researchers and artists. This review may also be relevant beyond artistic contexts, as wireless motion sensing is increasingly being investigated for movement sonification and rehabilitation [3,4].

For this paper, we compiled articles from the NIME and MoCo proceedings and performed a reference search using Google Scholar (March 2025, with no time restriction) with the following keywords: (1) *IMU* AND *music* AND *sound*, (2) *IMU* OR *accelerometer* AND *wireless* AND *music*, (3) *accelerometer* AND *music*. Each search returned more than 200 papers, from which we selected relevant works. It became clear that, apart from specific reviews on musical interfaces [5,6,7,8], the case of music and the performing arts is underrepresented in the literature on wearables and IMU applications. For example, the reviews in [9,10] mention only a single example of a musical application. We complemented our literature review with targeted searches related to the interface examples presented in this work.

This paper is organized as follows. First, we provide a historical overview of movement sensing in the performing arts, including IMUs and other technologies. Second, we describe IMU principles along with wireless transmission. Third, we present common IMU processing techniques typically applied in the performing arts. Fourth, we introduce a selection of hardware systems that have been used by artistic communities over the past decade. Finally, we conclude with a short discussion. Please note that all abbreviations and acronyms are listed at the end of the paper.

## 2. Movement Sensing in Performing Arts: Historical Perspectives

### 2.1. Capacitive Sensing and Sonar Systems

As already mentioned, the Theremin marked the beginning of using body gestures and movements without any tangible interface or instrument to produce sound and music. This opened up new forms of musical interaction, including with dance. An early milestone was the collaboration between Merce Cunningham and John Cage in 1965 for Cunningham’s piece *Variation V*, which employed a sound production system including theremins, tape recorders, and shortwave radio receivers [11]. Dancers also interacted with the sound by triggering light beam barriers and photocells connected to an electronic interface board that controlled the sound devices, all without physical contact with any artefact.

**Figure 1:**
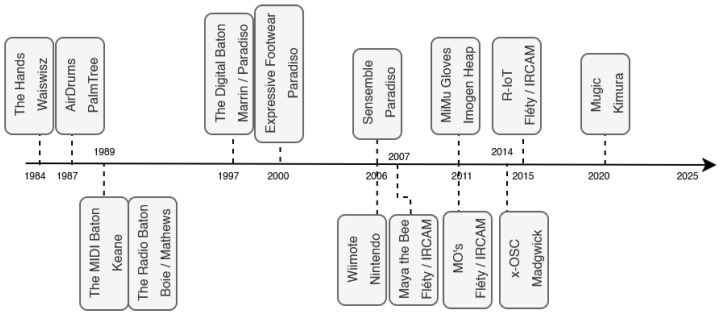
Timeline of Notables Movement-based Electronic Musical Instruments and Controllers since the 1980s.

As shown in Figure 1, several other gestural musical interfaces appeared in the 1980s, such as *The Hands* by Michel Waiswisz. Pioneered by Max Mathews, the *Radio Baton* (also known as the *Radio Drum*) is considered the precursor of a long line of hand-held devices used to control sound synthesis and music [12], based on capacitive sensing methods or ultrasound sonars [13]. Such interactive devices were further explored in the 1990s, with many stick controllers inspired by the conducting baton metaphor, communicating digitally with computers using the MIDI standard [14,15,16].

While some of these devices attempted to measure absolute positioning in space using electric field sensing or light, other techniques emerged in the 1990s, such as force-resistive or bending sensors. The *MIDI dancer*, which used radio transmission of bending sensor data for solo dance performance, is one of the earliest examples [17].

At that time, the techniques and hardware available for digitizing, encoding, and transmitting sensor data to a sound unit or computer lacked flexibility. As a result, performers and digital instrument designers were often forced to either adapt or hack existing controllers or interfaces to suit their musical needs, or to combine several interfaces or instruments [18]. By the late 1990s, musical controllers increasingly took the form of *ad hoc* instruments and electronic interfaces (Digital Musical Instruments or DMIs), including digitizers and encoders for protocols such as MIDI.

In addition to the proximity and non-contact sensing interfaces mentioned above, video-based systems emerged in the mid-1980s to capture full-body movement. Early hardware and software solutions included David Rokeby’s *Very Nervous System (VNS)*, which analyzed video streams for use in music, dance, and installations [19,20]; Tom Demeyer’s *Big Eye* (1995–2001), which converted video parameters to MIDI [21]; and Eyesweb [22]. Since then, other software environments have appeared, notably Jitter for *Max* (Cycling’74) (https://cycling74.com/products/jitter, accessed on 17 May 2025) and *Isadora* (Troikatronix) (https://troikatronix.com/, accessed on 17 May 2025). The commercialization of depth-sensing cameras such as Microsoft’s *Kinect* also enabled numerous performing arts applications. These systems are now increasingly being replaced by deep learning methods able to track skeletons with standard video cameras, such as *posenet* and *movenet* [23].

In this paper, we focus on another type of movement sensing: the Inertial Measurement Unit, which has played an increasingly important role in interactive systems for the performing arts, as described in [6].

### 2.2. Inertial Measurement Units

Inertial Measurement Units (IMUs) combine different sensors, such as accelerometers, gyroscopes, and magnetometers, to measure the attitude, orientation, and, in some cases, the displacement of a moving body—such as a gestural interface, a car, an airplane, or a spacecraft. In fact, these sensors have long been used in the aviation and robotics industries to measure angular position and attitude for manned and unmanned navigation. IMU technology has also been adopted for physical measurements, such as monitoring seismic activity or vibration.

Generally faster compared with video-based technologies, body and instrumental motion sensing using IMUs began in the late 1980s. For example, the *Airdrums* [24] from PalmTree Instruments used a clever arrangement of 2x6 mass–spring acceleration-based switches to trigger MIDI musical events generated by both linear and rotational acceleration. Keane’s *MIDI baton* [25] relied on a similar technique.

**Figure 2:**
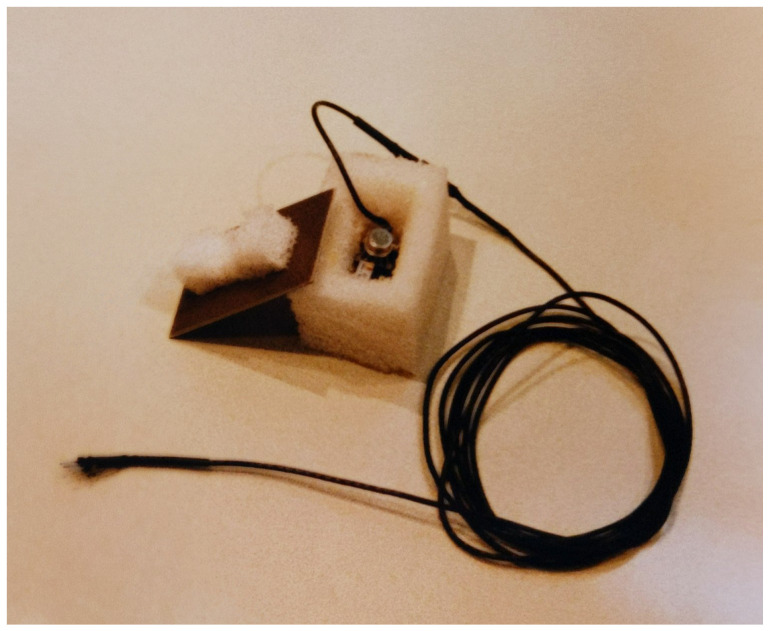
A ADXL50 accelerometer to be attached to the head of a violin for computer music score triggering—IRCAM 1996.

Early devices were built around bulky strain gauges and piezoelectric film or ceramics to measure mechanical deformation caused by acceleration. Miniature accelerometers emerged in the early 1990s thanks to Micro-Electro-Mechanical Systems (MEMS) technology. Analog Devices produced the first manufactured MEMS accelerometer in 1993: the ADXL50, a monolithic sensor with integrated conditioning that delivered an analog voltage representative of the applied acceleration [26,27]. The adoption of miniature inertial components quickly accelerated. The *digital baton* [28] was one of the first wired musical controllers to use MEMS accelerometers similar to those in use today. At IRCAM, the ADXL50 single-axis accelerometer was first used in 1996 in the context of an electroacoustic music piece [Figure 2]. The sensor was attached to a violin head so the performer could trigger musical events in the computer score of *Eluvion Etude* by Lucia Ronchetti [29]. The accelerometer’s analog signal was digitized and converted into MIDI information with an i-Cube sensor-to-MIDI interface [18].

In 2003, an interdisciplinary work group was created at IRCAM to further develop tools and instruments for the performing arts, including dance sensing technologies and augmented instruments—i.e., traditional instruments enhanced with additional sensing and interaction capabilities, similar to the hyperinstruments pioneered at MIT [30,31]. In particular, we developed a series of augmented violin sensing systems, initially with the ADXL202 [32], enabling the recognition of bow strokes without altering playing techniques or drastically modifying the instrument.

**Figure 3:**
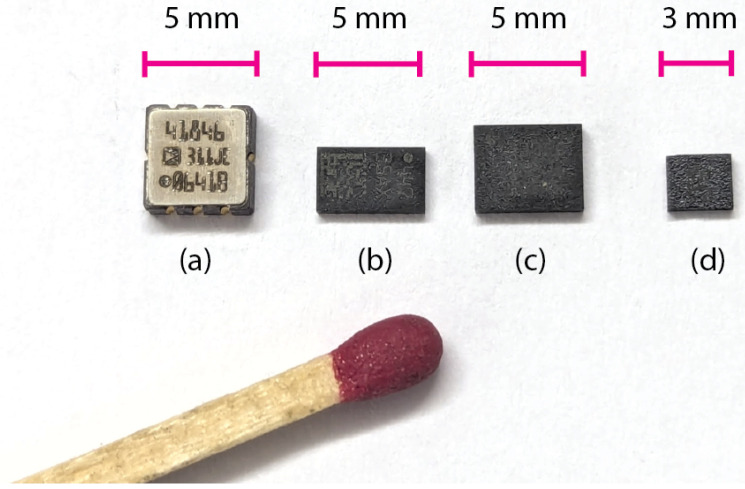
Size comparison of multi-axis MEMS accelerometers and packages (author’s collection). (**a**) ADXL202/2007—2× analog (**b**) LIS352/2013—3× analog (**c**) LSM330/2013—6× digital (**d**) LSM6D/2018—6× digital.

Later, the Bosch Sensortec SMB380 became the first compact 3-axis digital accelerometer on the market (2007), quickly followed by the ST LIS331. These marked the emergence of pre-digitized sensors, replacing the earlier analog MEMS accelerometers that required external digitization [Figure 3a]. From that point onward, MEMS continued to advance in miniaturization, reaching package sizes 3 × 3 mm or smaller [Figure 3d].

In the 2000s, IMUs began to be embedded in game controllers, most notably the *Wiimote* (see Visi et al. [5] for an extensive review of such gaming interfaces), as well as in smartphones. This contributed to the increasing availability of low-cost IMUs for a wide range of applications, including musical contexts. Their small size, robustness (compared with fragile bending sensors), reactivity, and low latency made them particularly well suited for real-time control of music and other media.

Specifically, IMUs provide:

- compact form factor (a few cm) and lightweight design suitable for attachment to a musical instrument or performer’s body (<20 g),
- robustness over time, making them compatible with touring conditions (bending sensors, by contrast, wear out quickly),
- low latency and low jitter, both essential for musical controllers (<10 ms).

In the following sections, we describe the different hardware and software implementations of IMUs applicable to the performing arts.

## 3. Inertial Sensing and Wireless Transmission: Hardware Principles

### 3.1. Inertial Measurement Unit (IMU)

As noted earlier, an IMU is a composite sensor generally made of accelerometer, and/or gyroscope and/or magnetometer. A MARG sensor (Magnetic, Angular Rate, and Gravity) is specifically an 9-Degree fo Freedom (DoF) IMU, including the 3-axis high-sensitivity magnetometer capable of measuring Earth’s magnetic field. Here, we focus on modern MEMS implementations: micro-electromechanical devices etched directly on a silicon wafer and packaged within a 3 × 3 mm IC, or smaller (see Figure 3).

MEMS accelerometers employ an interlaced comb structure with a central moving mass. The displacement of this mass changes the distance between the comb electrodes, resulting in capacitance variations, similar to the operating principle of condenser electret microphones.

MEMS gyroscopes measure angular velocity using the Coriolis effect. An oscillating mass in a rotating reference frame experiences a perpendicular Coriolis force. In MEMS gyroscopes, the oscillation is generated via a piezoelectric effect, and the Coriolis force is measured through capacitance changes in a comb electrode structure, similar to the MEMS accelerometers.

Finally, MEMS magnetometers in IMUs and electronic compasses often exploit magnetoresistive materials to measure weak magnetic fields, such as Earth’s (typically 50 µT). Resistance changes are converted into voltage variations via Ohm’s Law.

### 3.2. Data Acquisition and Transmission

**Figure 4:**
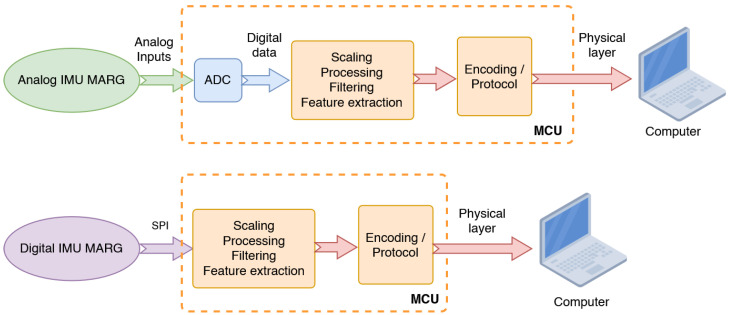
Compared topologies of acquiring analog or digital IMUs.

When provided as analog voltage-base sensors, IMU outputs must be digitized with an Analog-to-Digital Converter (ADC). When offered as digital sensors, data are acquired over a high-speed bus such as I^2^C or SPI [Figure 4]. The main acquisition loop of the microcontroller program should be timer-based with minimal drift to preserve alignment over time. In cases involving CPU-intensive algorithms, acquisition can be delegated to DMA to automate either the ADC or SPI transfers. After scaling, filtering, and optional fusion or feature extraction, IMU data are ready to be streamed to a computer. Modern digital IMUs typically provide 12–16-bit resolution, with some high-end models achieving even higher resolutions.

Embedded digitization allows pre-amplification and gain control at the MEMS cell, ensuring excellent digital mapping and signal-to-noise ratio (SNR) when higher ranges are required, such as for percussive impacts. Sensor data timestamping and synchronization can be achieved using computer network NTP techniques, either via standard servers or local implementations [33].

Cook’s design principles for performing arts systems, published in 2001, stated that wired connections were preferable to wireless ones [2]. He revisited his recommendation in 2007 in light of significant improvements in wireless technology, including the adoption of IEEE standards such as 802.11 (Wi-Fi) and 802.15.4 (Zigbee). Initial Cook’s criticism of wireless systems was well justified at the time, since early miniature digital radio transmitters based on ASK (AM) or FSK (FM) operated on only one or two frequency channels in the Instrumentation, Scientific, and Medical (ISM) radio band. These systems provided little to no data scheduling, which prevented the simultaneous use of multiple sensor units in the same space due to RF spectrum interference. Nevertheless, wireless digitizers were developed [34,35] using these transmitters as a foundation, but their practical use in live performance contexts remained limited, until the ISM band was extended to 2.4 GHz. This enabled multiple channels, greater spectrum availability, higher bandwidth, and several physical-layer implementations such as Bluetooth, Zigbee, and Wi-Fi.

**Figure 5:**
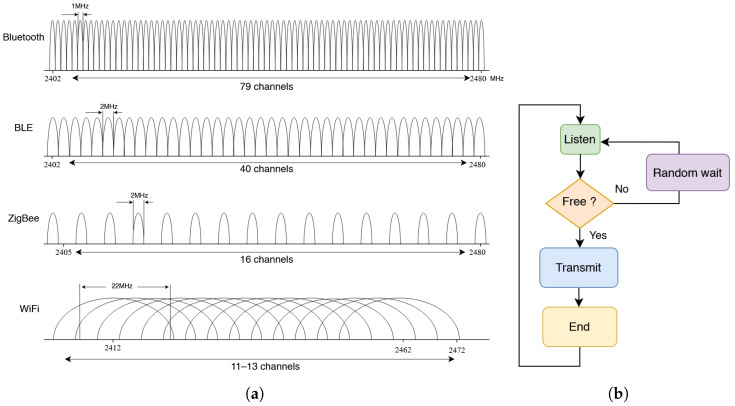
(**a**) 2.4 GHz spectrum of the ISM band with channel allocation for Bluetooth, BLE, Zigbee (IEEE 802.15.4), and WiFi (IEEE 802.11) [38]—(**b**) simplified flowchart of CSMA/CA transmission.

The early modules mentioned also lacked true packet radio functionality, which at best had to be implemented within the host microcontroller—already responsible for both digitization and data processing. The successful use of wireless gestural data indeed relies on CSMA/CA (Carrier Sense Multiple Access with Collision Avoidance). This technique, derived from CSMA/CD (Carrier Sense Multiple Access with Collision Detection) in the Ethernet implementation of OSI layers 1 and 2, allows transmitting stations to access the medium only when permitted, without requiring a fixed TDMA (Time Division Multiple Access) schedule. This approach anticipated the adoption of radio modems. A packet radio using CSMA/CA provides a stochastic mechanism that prevents transmitters from phase-locking on a single channel. It does so by listening to the carrier to ensure it is free before transmitting, or by waiting a random interval if the channel is occupied. By combining random backoff delays with a listen-before-transmit algorithm, this method establishes a self-regulated network with higher bandwidth efficiency compared to simplex or TDMA systems [36,37] [Figure 5].

In designing our sensor interfaces, and specifically our wireless IMU modules, we opted for CSMA/CA-based packet radio components [39,40]. We targeted standards such as Wi-Fi and Zigbee to ensure native compatibility with most computer platforms and operating systems, while maintaining overall low latency across the remaining OSI layers before reaching the final application, such as a Digital Audio Workstation (DAW) [40].

### 3.3. WiFi Versus Bluetooth

Wi-Fi has generally been preferred to Bluetooth for stage performances. First, Wi-Fi was designed as a wireless extension of Ethernet, which is inherently structured as a scalable network supporting multiple transmitters. Second, Wi-Fi benefits from native implementation of OSI layers 5 and 6 within most operating systems, improving processing efficiency and reducing latency. By contrast, Bluetooth has long relied on separate software stacks, adding overhead. Although Wi-Fi and Bluetooth operate in the same frequency band, Bluetooth was originally organized as a peer-to-peer protocol with a long timeout in case of connection loss. Reconnection could take 10–30 s, making reliable use in live performance nearly impossible.

Early Bluetooth versions (1 and 2) relied on channel hopping to avoid interference, which introduced gaps in throughput. Bluetooth Low Energy (BLE) improved on this with adaptive channel hopping and isochronous transfers, allowing multiple peripherals or stations to connect to a single host. It is worth noting that BLE 5 offers significantly better wireless transmission than earlier Bluetooth versions and can be suitable for our use cases.

Nevertheless, Wi-Fi still provides wider channels, enabling higher data rates. Wi-Fi physical layer (PHY) supports nominal data rates ranging from 11 to 300 Mbit/s, greatly surpassing BLE in raw performance. This should, however, be considered alongside the practical performance of Wi-Fi modules or Wi-Fi–equipped MCUs. For example, the ESP32 MCU achieves an effective throughput of around 20 Mbit/s, which still allows larger packets—and therefore more sensor data—to be transmitted at each interval, with lower overall latency for a given payload.

In summary, from our experience in the performing arts, we generally prefer Wi-Fi over BLE for two key reasons: (1) Wi-Fi allows scanning of the RF spectrum in a given venue and manual selection of a less congested channel—flexibility not available with BLE; and (2) Wi-Fi offers higher nominal transmission power and longer range, making it more reliable for covering large performance stages.

### 3.4. Protocol and Data Transmission

For wireless transmission of sensor data in live performance and DMIs, two protocols are commonly used: Open Sound Control (OSC) and MIDI-over-Bluetooth (MIDI-BLE), which will be described in more detail in the following section. Each protocol depends on its particular physical layer. OSC offers a flexible protocol and syntax across multiple carriers (UART, wired Ethernet, Wi-Fi), operating at the OSI presentation layer (Layer 5). MIDI-BLE is standardized within Bluetooth Low Energy and operates at the Generic Attribute Profile (GATT) layer.

#### 3.4.1. Sample Rate and Resolution Considerations

Typical sample rates for continuous gestural inputs range from 100–400 Hz, depending on the application and controlled media [41], with latency requirements below 10 ms. Nevertheless, it is important to note that computer music software implements real-time *control* of sound synthesis at rates generally equal to or below 1000 Hz, typically around 50–200 Hz. For this reason, computations requiring higher sampling rates should be performed at the microcontroller level, while wireless communication is set to lower rates (<1000 Hz, typically 50–200 Hz).

The Musical Instrument Digital Interface (MIDI) protocol was developed in the early 1980s to represent, encode, and transmit musical information from controllers—such as MIDI keyboards—to synthesizers. For decades, MIDI has been used to build DMIs and controllers [42,43]. Despite its popularity in the DMI context, the MIDI 1.0 specification presents several limitations. Traditional MIDI operates over a current-loop UART at 31,250 bps offering a maximum throughput of 1041 messages per second on a single link. Under these constraints, the performance of gesture encoding degrades rapidly as the number of transmitted sensors increases, a factor often referred to as the real-time sensors polyphony. In practice, wired MIDI is limited to around 10 sensors on a single link when maintaining an upper bound of 10 ms latency.

Wireless MIDI was implemented over Bluetooth 1.0 and 2.0 by transmitting MIDI-formatted messages over an SSP profile emulating a serial port at the MIDI baud rate, instead of the usual UART computer baud rates. However, the SSP profile lacked synchronization tokens to packetize MIDI chunks, resulting in transmission gaps and lags due to unpredictable latency when splitting MIDI messages. Nevertheless, MIDI-BLE remains practical and energy-efficient for short-range, solo-performer wireless controllers connected to a computer, even if limited to the standard 7-bit MIDI resolution [44]. Higher resolution can be achieved by combining LSB and MSB messages, but this comes at the cost of reduced data rate and increased latency, even when using MIDI’s running status optimization. These limitations naturally led us to migrate our gestural sensor interfaces toward the Open Sound Control (OSC) protocol, which supports transmission up to 64-bit floating-point numerical values over long distances (wired or wireless).

Combining Wi-Fi and Open Sound Control has proven to be a reliable solution for concert and multi-user contexts, as demonstrated in the field study by Mitchell et al. [45]. It also achieves some of the lowest latencies for given payloads or throughput in practical conditions using MCU-based hardware [46].

In summary, OSC is generally preferred for low-latency, high-throughput applications, partly due to its higher native transport resolution. MIDI-BLE remains popular for straightforward backward compatibility with native MIDI software, although it offers lower resolution.

#### 3.4.2. Efficiency

Transmitting over a physical carrier requires protocol encoding to structure the IMU data payload. Depending on the OSI model layer at which integration is performed, complying with a given protocol can be more or less costly. This cost directly affects rate efficiency, or the useful data throughput, defined as:

$$Efficiency\left(\%\right)=\frac{Useful\;Data\;Payload}{Total\;Payload}\times 100$$

**Figure 6:**
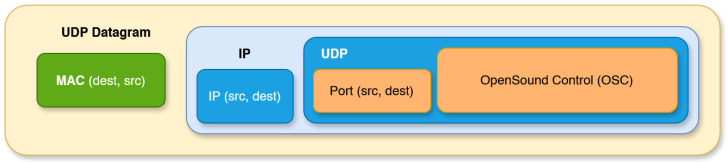
Structure of an OSC package encapsulation within a UDP datagram.

In the case of the OSC protocol, sensor data are encoded in UDP packets or *datagrams* [Figure 6].

These packets include the Ethernet MAC layer preamble, followed by the IP layer, and finally the UDP payload. A datagram with no payload requires 46 bytes of structure, including MAC addresses, IP addresses, and UDP ports. Efficiency increases significantly once several sensor data are packed into the same datagram, whose maximum size (MTU, Maximum Transmission Unit) defaults to 1536 bytes but may vary. As a numerical example, consider an OSC message beginning with its address followed by 9 × 16-bit raw IMU values (3-axis accelerometer, 3-axis gyroscope, and 3-axis magnetometer). Assuming the MAC/IP/UDP base packet structure is 46 bytes, we obtain a theoretical, header-level efficiency of:

$$Efficiency\left(\%\right)=\frac{64}{\left(64+46\right)}\times 100=58\%$$

Therefore, a trade-off must be considered in UDP packet construction: aggregating multiple contents within a single packet improves efficiency, but the packet forging time required by the hardware must also be taken into account. While negligible on computers, this cost can be significant for embedded electronics. Our recent wireless Wi-Fi IMU modules achieve OSC packet forging times in the range of 150–300 µs for a data payload of 20–30 numerical values, resulting in efficiencies of 72–78% while remaining below the standard MTU. When grouping data within a single datagram, subset parsing and routing are preserved thanks to the OSC bundle structure [47].

## 4. IMU Processing for Performing Arts

From a user perspective, gesture-sensing technologies can be divided into two categories. First, some sensors provide output data that are directly interpretable by the user because the data correspond to familiar physical quantities [48]. Typical examples include distance/proximity sensors, pressure sensors, and bending sensors. Second, other sensors, such as IMUs, require pre-processing to extract meaningful data [49]. Accelerometers represent such a typical example: as mentionned, the reported raw data correspond to the deviation of a mass–spring system, not to acceleration as defined in classical kinematics. Raw accelerometer data are thus difficult to interpret intuitively because they combine two components: (1) orientation relative to gravity (see Appendix A) and (2) linear dynamic acceleration. Moreover, dynamic acceleration is itself a quantity that users are less familiar with compared to spatial coordinates (x, y, z) or velocity-based parameters.

Therefore, the raw IMU data must generally be processed before being used in concrete applications in performing arts. We focus here on cases where the IMU is analyzed in real time for human–machine interaction scenarios, which imposes additional constraints compared with offline analysis, as found in many IoT applications.

Note that here we consider the use of cost-effective IMUs (typically costing a few dollars) in the performing arts, which are intended to measure orientation or rotational movements rather than displacement. Accurate displacement measurement would require high-end IMUs (tens of thousands of dollars), which are generally unaffordable in performing arts contexts. For this reason, the use cases described in this paper do not include inertial navigation (i.e., computing displacement from acceleration), which is feasible only with high-end IMUs or by combining IMUs with vision-based technologies.

In performance arts, the relationship established between the sensors dataflow and the parameters controlling the output media (sound, visuals, lighting, etc.) is refereed to the *mapping* process. Programming this mapping is generally handled by artists/designers using dedicated software. With adequate processing, IMUs can be used in several mapping strategies such as:

- Triggering events such as sound samples or MIDI events. This typically requires computing filtered acceleration intensities (see Section 4.2.1) with an onset detection (see Section 4.2.3), or using zero crossing in gyroscopes.
- Continuous “direct” mapping using the orientation (see Section 4.1.4), angular velocities from gyroscopes, or filtered acceleration intensities.
- So-called “indirect” mapping strategies using machine learning (software examples in Section 4.3). This often involves concatenating parameters of the processed parameters we mentioned (e.g. filtered acceleration intensities, orientation, and angular velocities).

For concrete artistic applications, we refer readers to [50] on sound triggering and continuous direct mapping and [32,51,52] on indirect mapping using machine learning.

We describe below different calibration, pre-processing and motion descriptors computation, generally necessary with IMUS.

### 4.1. Raw Data Processing

Raw IMU data are generally processed through multiple steps, as described in the following sections.

#### 4.1.1. Sensitivity and Resolution Adjustments

Modern IMUs are mostly “digital sensors”, performing onboard sampling and digitization before exporting data over high-speed buses such as I^2^C or SPI. The same interface is also used to configure the physical behavior of the MEMS unit, including bandwidth, sensitivity, scale, and resolution. Temperature drift of IMU readings should ideally be taken into account in computing specific features, especially for orientation calculation using gyroscopes.

The sensor’s output amplitude depends on its internal scaling and the executed gesture. To preserve gesture nuances, appropriate resolution and scaling must be configured. For example, for mid-air gestures, a high gain (+/−2 g) is typically suitable for accelerometers. This sensitivity is achieved via an internal analog amplifier and gain, mapped to the ADC resolution (e.g., 16 bits) to maximize gesture resolution. Lower sensitivity is needed for high-dynamic movements, such as bowed string techniques [32,53] or percussion gestures, where up to 8 g acceleration may be required.

#### 4.1.2. Sampling Rate Conversion

Sample rate conversion may be necessary to achieve the minimal latency required by the application. For instance, triggering sound from acceleration onset detection typically requires latency below 10 ms to maintain the system responsiveness needed for rhythmic musical contexts, whereas higher sampling intervals of up to 20 ms may be acceptable for continuous musical interactions based on slow, fluid movements. Because some feature extraction can be CPU-intensive, sensor down-sampling may also be beneficial in these cases.

Additionally, sample rate conversion may be needed to simplify real-time processing when sensors do not share identical output data rates (ODR). In particular, magnetometers generally have lower ODRs compared with gyroscopes or accelerometers.

#### 4.1.3. IMU Calibration

Like most electronic devices and sensors, IMUs feature both a static offset and floor noise:

OutputValue(n)=ActualValue(n)+Offset+Noise(n)

The calibration phase involves determining the offset as accurately as possible in order to nullify it and to prevent or reduce drift during mathematical integration.

**Gyroscope calibration:** A still gyroscope exhibits a voltage offset in the measured angular speed. To obtain the most accurate approximation of this offset, the sensor should be held completely still while the mean background noise is computed for all three axes simultaneously. The result is stored, and the offsets are then subtracted from the live values received from the gyroscope.

**Accelerometer calibration:** An accelerometer primarily exhibits a plane alignment offset, defined by the angular deviation between the MEMS plane and the Earth’s reference for measuring the gravity vector. This alignment issue can have multiple causes:

- Voltage offset in the signal conditioner or the sensor’s internal ADC, as it is also typically found in gyroscopes.
- MEMS engraving offset on the silicon.
- Misalignment of the sensor’s IC casing on the PCB.
- Geometric offset or mechanical misalignment of the sensor within its housing.

To measure offsets, the IMU should be slowly rotated so that all six faces of the board are successively oriented vertically to experience gravitational acceleration. To ensure that gravity dominates over acceleration components caused by in-hand manipulation of the board and to avoid abrupt changes or high-frequency noise, the incoming acceleration data streams must be heavily smoothed using a low-pass filter. Consequently, it can take several seconds for the calibration parameters to stabilize at their correct values. A recommended approach is to provide users with visual feedback indicating when a parameter has become sufficiently stable (i.e., within a pre-set threshold). After this process, static acceleration values, resulting from gravity, should range between −1 g and 1 g on all three axes. For each axis, the minimum and maximum accelerometer values are stored as calibration parameters and used to scale the output stream accordingly.

**Magnetometer calibration:** This is the most complex calibration to perform. To enable IMU data fusion or to extract compass information, the magnetometer must be centered. The magnetometer measures the projection of the Earth’s electromagnetic field onto the sensor’s planes, which can include non-zero magnetic fields depending on the location and surrounding objects, such as loudspeakers. To center the readings, the IMU is slowly rotated along all axes to measure the minimum and maximum values of the detected magnetic field, and the offsets for each axis are calculated as follows:

$$offset=\frac{\left(max+min\right)}{2}$$

The stored offsets are also referred to as hard-iron calibration offsets or biases. Additionally, the linear response of each magnetometer can be affected and distorted by local magnetic or ferrous materials, such as PCB traces, shielding, or the battery. These distortions are called soft-iron perturbations and usually induce cross-axis nonlinearities, resulting in a non-spherical distribution of magnetic measurements. Compensation for soft-iron distortions is generally achieved using a cross-correlation matrix with eigenvalue decomposition or an ellipsoid-fitting algorithm to reshape the magnetometer measurements into a sphere. This process can be performed during calibration by spinning the IMU along its axes while logging magnetic data, or by running a live calibration using Kalman filter techniques [54].

#### 4.1.4. Orientation Algorithms

The 3D angular position of the sensor in space, also referred to as the Attitude and Heading Reference System (AHRS), can be derived by fusing either 6D IMU data (3D acceleration and 3D angular velocity) [55,56] or 9D IMU data (3D acceleration, 3D angular velocity, and 3D magnetic field). Data fusion in these cases is also called an orientation filter.

The 3D angular orientation is generally expressed using Euler angles, a rotation matrix, or quaternions, computed using a fusion algorithm such as Mahony’s complementary filter [57] or Madgwick’s gradient descent [58]. Some integrated IMUs, such as the Bosch Sensortech BNO055, directly embed computation to provide AHRS parameters. The online reference by Mario Garcia (https://ahrs.readthedocs.io/, accessed 17 May 2025) provides a comprehensive review of algorithms along with Python 3.6 code implementations.

Several other attitude representations, which do not include the heading angle (relative to magnetic North), can also be computed—for example, the orientation relative to Earth’s local tangent plane (also called gravity). This can be expressed as the projection of the gravitational acceleration vector onto the X and Y axes, which is valid only when dynamic acceleration is null [59], as described in Appendix A. In other cases, a complementary filter combining accelerometers and gyroscope can be efficiently implemented.

#### 4.1.5. Standard Filtering

Filtering can be achieved using classic signal processing techniques, such as digital low-pass, high-pass and band-pass filters. Typically, the use of one-pole or bi-quad filters is efficient in real-time applications. For example, applying a band-pass filter at 10 Hz (Q = 1) to accelerometer data is generally effective for mid-air gestures, as it filters high-frequency noise and removes orientation offsets in a single step.

Median filters can also be useful for reducing noise artifacts and discontinuities in IMU data. This is done by applying a sliding window to the input signal and outputting the middle element of the resulting array. It should be noted that any filtering process introduces latency, which must be considered when selecting a filter (type, architecture, model).

### 4.2. IMU Analysis and Feature Extraction

For a comprehensive review of motion analysis using IMUs, we refer the readers to [60]. Additionally, Freire et al. provide an interesting study comparing the use of IMUs with Optical Motion Capture [61]. We focus below on basic parameters that can be easily extracted for designing gesture-sound mappings. We draw attention to simple yet effective processing techniques, which proved especially useful in our applications, but that are rarely described in the literature.

#### 4.2.1. Movement Intensities Derived from Acceleration Computation

The orientation filters, as previously described, provide parameters that are easily understood by designers and artists for building real-time gestural controls. In particular, the orientation angles are simple to use. In contrast, linear acceleration is less intuitive, as we are more accustomed to parameters such as velocity. For this reason, it is generally useful to derive quantities that can be intuitively apprehended by artists and designers.

To achieve this, we derive different possible ‘filtered acceleration intensities’ *I* from the raw acceleration values (either along a single axis or using the full 3D vector): first, applying a derivative process (which removes the slow orientation offset of accelerometer data) and then a windowed (or weighted) integration process on either (1) the absolute value (Equation (1)) [62] (possibly averaging over axes), or (2) the squared value of a given axis or the vector norm (Equation (2)). In some cases, retaining only the positive part of the derivative can be effective for segmentation tasks along a specific movement direction.

(1) $$\mathrm{Option}\;1:\;I_{1}\left(t\right)=\frac{k_{1}}{\Delta }\int_{t-\Delta }^{t}\left|\frac{da}{d\tau }\right|d\tau$$

(2) $$\mathrm{Option}\;2:\;I_{2}\left(t\right)=\frac{k_{2}}{\Delta }\int_{t-\Delta }^{t}{∥\frac{da}{d\tau }∥}^{2}d\tau$$

where a(t) is the raw accelerometer signal (including gravity), and Δ is the duration of a moving time window.

**Figure 7:**
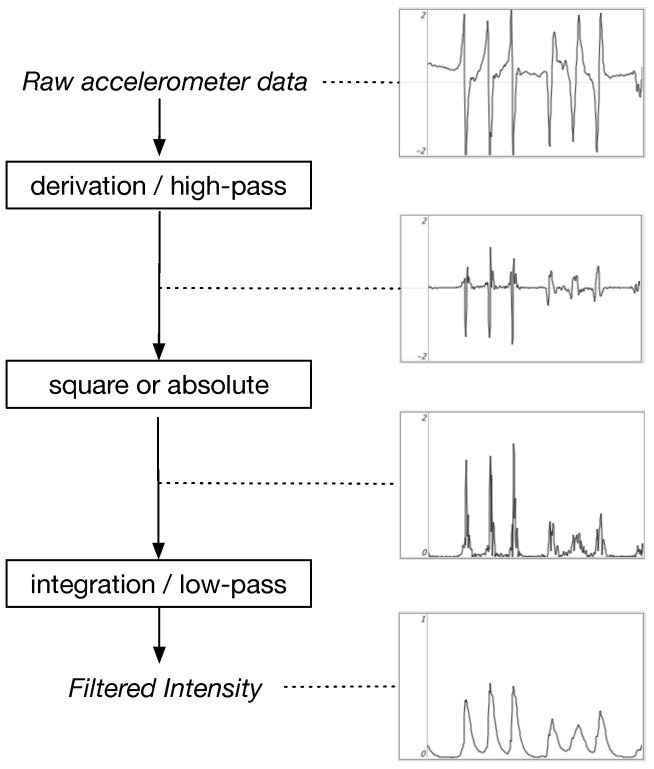
Processes to derive an acceleration-based filtered intensity. With a framerate of 100 Hz, the derivative can be computed, for example, using a linear regression over 3 points (or a high-pass bi-quad filter with $f_{c}=4$ Hz and Q=0.45), and a low-pass bi-quad filter with $f_{c}=10$ Hz and Q=0.45).

The implementation of this formula can be performed using simple IIR filtering techniques, as indicated in Figure 7. In practice, these intensities are also modified using non-linear functions, for example, a power function with an exponent of 0.5 or 0.25, which emphasizes lower intensities. Unlike the raw acceleration value, these intensities are always positive and equal to zero when there is no movement.

To interpret these intensities, it is worth to note that, when moving freely, we tend to minimize jerk, i.e. the derivation of the acceleration, avoiding thus steep accelerations. As initially described by Flash and Hogan, spontaneously moving from an initial to a final position in a given time Δ, human trajectories typically minimize a cost function following Equation (2) [63,64]. Therefore, the quantities we propose reflect “intensities” of intentional, marked movements, as found for example in beating gestures, or interacting with surfaces and objects.

#### 4.2.2. Stillness Computation

It is useful to define "rest states" in interaction design, which we refer to here as *stillness* (i.e., no movement). There are several approaches to computing this quantity from the IMU. The most straightforward methods involve defining a threshold on the filtered acceleration (or acceleration without gravity) or on the gyroscope norm values. However, these approaches can be sensitive to calibration and offset changes, particularly due to temperature variations. An possible alternative, for human movement, is to compute the stillness state using a threshold on the quantity $\sqrt{{\left(w_{y}-w_{z}\right)}^{2}+{\left(w_{z}-w_{x}\right)}^{2}+{\left(w_{x}-w_{y}\right)}^{2}}$, which is norm of the vector product $∥\vec{w}\wedge \vec{n}{)∥}^{2}$, where $\vec{w}$ is the 3D angular velocities from the gyroscope, and $\vec{n}$ the directional vector $\vec{n}=\left(1,1,1\right)$. This measure is invariant to equal shifts in all three dimensions of the gyroscope. Its advantage is that it is highly sensitive to small IMU movements while remaining independent of potential gyroscope offset drift over time.

#### 4.2.3. Onset Detection

An efficient approach for detecting movement onsets is to set a threshold on the difference between a data stream and its filtered values using a median filter. The latency of onset detection directly depends on the median filter’s window size. Adding a gate that prevents re-triggering within a given time window improves the stability of onset detection. This approach generally works effectively on the filtered intensity described in Section 4.2.1.

#### 4.2.4. High-Level Motion Descriptors

As described by Camurri and coworkers [65,66], it is generally necessary, when building interactive media systems, to derive so-called *high-level motion descriptors*. For example, metaphors and playing techniques are used to co-design meaningful interaction paradigms with designers and artists [50,67,68,69,70]. These can involve gestures such as *fluid* movement or *shaking*. We refer the reader to Larboulette and Gibet [71] and Niewiadomski et al. [72] for a series of computable *movement qualities*. Moreover, Visi and Tanaka [52] provide a review of interactive machine learning techniques that can be applied to musical gestures.

### 4.3. Software Toolbox of Interactive Use Cases

Below, we present some of the software modules used for real-time IMU data processing (in alphabetical order):

- The *Digital Orchestra Toolbox* is a open-source collection of Max modules for the development of Digital Musical Instruments, including usefuel resources for IMU processing (https://github.com/malloch/digital-orchestra-toolbox, accessed on 17 May 2025).
- *EyesWeb*, by Antonio Camurri and colleagues [22], is a patch-based programming environment for body movement–computer interaction and visual arts (http://www.infomus.org/eyesweb_eng.php, accessed on 17 May 2025).
- The *Gesture Recognition Toolkit* (GRT) is a cross-platform, open-source C++ machine-learning library specifically designed for real-time gesture recognition (https://github.com/nickgillian/grt, accessed on 17 May 2025). In addition to a comprehensive C++ API, the GRT also includes an easy-to-use graphical user interface.
- The *Gestural Sound Toolkit*, using *MuBu* (see below), is a package that provides objects to facilitate sensor processing, triggering, gesture recognition [73]. It contains examples of gesture to sound mapping, using direct or indirect methods with Machine Learning. The toolkit accepts diverse data inputs, including wireless IMU from the R-IoT module (Section 5.3) or from smartphone inertial sensors using the *Comote* app (Section 5) (https://github.com/ircam-ismm/Gestural-Sound-Toolkit, accessed on 17 May 2025).
- The *Libmapper* library is a system for representing input and output signals on a network and for allowing arbitrary “mappings” to be dynamically created between them (http://idmil.org/software/libmapper, accessed on 17 May 2025).
- *MuBu* is a package for *Max* (Cycling’74) that contains several objects for real-time sensor analysis, including filtering (such as the accelerometer intensities described in Section 4.2.1) and gesture recognition algorithms that can be trained with one or a few examples provided by the users [51] (https://forum.ircam.fr/projects/detail/mubu/, accessed on 17 May 2025)
- *Soundcool* is an interactive system for collaborative sound and visual creation using smartphones, sensors and other devices (http://soundcool.org). See Dannenberg et al. [74] for examples in music education.
- the *Wekinator* is a stand-alone applications for using machine learning to build real-time interactive systems (http://www.wekinator.org/, accessed on 17 May 2025).

## 5. Recent Systems and Devices Examples

We present in this section several wireless IMUs used for artistic and live performances or for gestural research, which have been employed over the last ten years.

As previously mentioned, game interfaces [5] and smartphones often embed IMUs, which can be streamed using applications such as (all links accessed on 17 May 2025):

- *Comote* (https://apps.ismm.ircam.fr/comote)
- *gyrOSC* (https://www.bitshapesoftware.com/instruments/gyrosc/)
- *MediaPipe* (https://chuoling.github.io/mediapipe/)
- *MotionSender* (http://louismccallum.com/portfolio/wekinator-motion-app)
- *touchOSC* (https://hexler.net/touchosc)

We report below on hardware devices including IMUs with streaming capabilities for general-purpose applications (in alphabetical order). Concerning specific applications, see for example the recent MetaBow [53], with a wireless IMU sensor embedded in a violin bow.

### 5.1. Mi.Mu Gloves

Mi.Mu gloves combine composite IMUs with gloves and finger flexion sensors, developed in collaboration with artist Imogen Heap and initially based on the X-OSC unit (9-DoF MARG) mentioned above. The system consists of a pair of gloves, each equipped with a wrist-mounted housing for the wireless IMU and sensor hub. Designed for live computer music control, they allow for bi-manual expressivity and the use of the body as an instrument. Specific software called Glover is provided to create mapping strategies between hand postures and sound parameters, controlling software such as Ableton Live. Dimensions (estimated from the 18650 Li-ion battery): 70 × 60 × 20 mm. (https://www.mimugloves.com, accessed on 17 May 2025).

### 5.2. MUGIC

MUGIC^©^ is a wireless IMU developed by composer and violin player Mari Kimura to modulate and control music with gestures like bowing motion. It uses Wi-Fi and Open Sound Control to stream data from the Bosch Sensortech BNO055 9-DoF IMU. It streams raw and fused data at rates between 40 and 100 Hz, with a runtime of approximately 2 h. Example patches are provided to link and map data to musical content within Max/MSP or Ableton Live. Dimensions: 40 × 20 × 10 mm, weight: 18 g (with case). (https://mugicmotion.com/, accessed on 17 May 2025).

### 5.3. R-IoT

**Figure 8:**
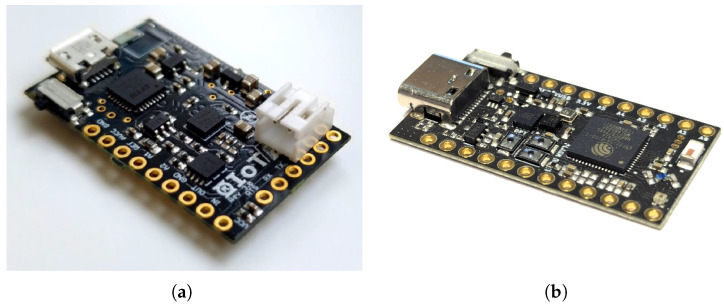
(**a**) The R-IoT wireless IMU version 2 (**b**) The R-IoT wireless IMU version 3.

The R-IoT is a 9-DoF Wi-Fi/OSC IMU (MARG) developed at IRCAM for building DMIs and for research purposes (Figure 8). The latest version, R-IoT v3, uses the ESP32-S3. In addition to streaming raw sensor data and the Madgwick orientation filter [58], it features additional analog and digital inputs, a barometer, as well as a spare I^2^C bus and a TTL serial port for additional controls, which can be hubbed and streamed at a rate of 200 Hz.

The R-IoT board is provided with open-source firmware written in C++ and a set of Max/MSP patches to receive and process sensor data to extract features, as presented in Section 4 (https://github.com/ircam-ismm/riot-v3/, accessed on 17 May 2025). Features extraction can also be computed on board using C/C++. From version 3, data streaming uses a uniform and standardized OSC syntax based on the W3C Device Orientation and Motion taxonomy (https://www.w3.org/TR/orientation-event/, accessed on 17 May 2025). Raw dimensions: 34 × 18 × 3.5 mm (2 g, card only)—2–8 h runtime.

### 5.4. SOMI-1

SOMI-1 is a wrist-worn 9-DoF IMU (MARG) that streams data over BLE 5 to a dedicated MIDI-USB receiver and hub. In addition to the usual raw sensor outputs, it provides an orientation filter and onboard data fusion. SOMI is coupled with scaling and mapping software, an Ableton Live editor, and a companion smartphone music app. Dimensions: 40 mm outer diameter; weight: 9.4 g. (https://instrumentsofthings.com, accessed on 17 May 2025)

### 5.5. Wave

The Wave is a MIDI-BLE wireless IMU in the shape of a ring, developed by Genki. It contains an attitude sensor fusing data from a 3D accelerometer and 3D gyroscope, streaming pitch and roll angles as MIDI controllers over BLE (2D, Mahonny-type orientation filter). Additionally, it provides three switches and a nine-LED display for visual feedback. Wave is bundled with Softwave, a MIDI mapping software designed to control a synthesizer via hand or finger gestures (timbre, vibrato, pitch bend, reverb). Dimensions: 28 × 21 × 24 mm; runtime: 8 h. (https://genkiinstruments.com/wave, accessed on 17 May 2025)

### 5.6. WiDig + Orient4D

The *WiDig* is a Wi-Fi/OSC or MIDI-BLE sensor digitizer created by Infusion System, the company behind the I-Cube system, an early sensor-to-MIDI converter for custom DMIs and art installations. Various sensors can be plugged in and digitized, including the Orient4D sensor, a 9-DoF device providing raw acceleration, gyroscope, and magnetic vectors, as well as AHRS, at a maximum update rate of 100 Hz. WiDig is supplied with tutorials and EditorX, a program for configuring the unit and performing preprocessing operations such as filtering, scaling, or feature extraction. Dimensions: 85 × 26 × 18 mm; weight: 20 g. (https://infusionsystems.com, accessed on 17 May 2025).

### 5.7. x-IMU3

This wireless IMU is the third iteration of the former X-OSC device developed by Sebastian Madgwick, author of the widely used Madgwick orientation filter [58]. It features a composite 9-DoF IMU (MARG) plus an additional high-g accelerometer and streams 15 data channels, including AHRS angles, over Wi-Fi or Bluetooth 2.0 at a 400 Hz update rate. Although primarily targeted at gestural research applications, it can also be used for live performance mapping and musical interaction. Max (Cycling’74) as well as C, C++ and Python examples and API are provided to facilitate the use and mapping of received data. Dimensions: 54.6 × 48.49 × 13 mm (https://x-io.co.uk/x-imu3/, accessed on 17 May 2025).

### 5.8. Summary

The wireless IMU modules presented in the previous sections are designed to stream body movement data with relatively low latency and sample rates suitable for the real-time requirements (≥100 Hz) of musical applications, which constitute their primary target. The technical aspects are summarized in [Table 1], including the sensor type, samplerate, streaming protocol, and programmability. While the x-IMU3 is more oriented toward research applications and largely exceeds typical sample rate expectations, it can still be used with computer music software via a proxy application programmed with its provided software API (for example, an OSC bridge).

Most devices feature closed-source firmware but provide complementary proxy software designed to map, route, and extract motion features for use in the final application. Concerning data fusion and calibration, we observe two different types of devices in the list presented. On one hand, items such as MUGIC and WiDig use a meta-IMU with internal fusion and a proprietary algorithm (Bosch BNO055). On the other hand, systems like x-IMU3 and R-IoT use a discrete set of sensors and perform data fusion and computation within the MCU. Embedded fusion provides a more user-friendly calibration process that runs autonomously but does not allow for adjustments. MUGIC reports the calibration state of the meta-sensor in its OSC stream, and calibration is automatically performed each time the unit is powered on. Systems with dedicated fusion, by contrast, offer an on-demand calibration process that can be performed each time magnetic environment changes, such as when performing in a new location.

Overall, these devices are complementary, forming a rich ecosystem of IMU modules for controlling sound and media through movement or instrumental gestures, each accommodating different design choices and constraints. They cover a broad spectrum from beginners to expert DMI designers makers and researchers.

**Artistic application examples and references**

We provide below a selection of references and artistic applications for each of the listed modules and present use cases.

- **Mi.Mu Gloves:** Controlling virtual musical instruments with gloves-based interface and mid-air music performance [75,76].
- **MUGIC**: Violin and augmented instruments [77], mid-air gestures [78].
- **R-IoT:** Mid-air gesture and virtual percussion [79], conducting electronic music [80], capturing bowing gestures [81].
- **SOMI-1:** Dance performance with sensors [82], motion capture of dance [83].
- **Wave:** Performer-controlled interactive audio system for Opera singers [84] (also using MUGIC).
- **WiDig + Orient4D:** Tangible interface controlling audiovisual contents [85].
- **x-IMU3:** Motion sensing for creative industries [86]. See also [87] for applications of their earlier hardware version.

## 6. Discussion and Conclusions

As pointed out by Medeiros and Wanderley [6], IMU systems have seen growing interest in musical applications, following the trend of their adoption in consumer electronics, including smartphones and IoT devices. Compared with popular use cases such as personal navigation or home automation, movement-based control in the performing arts represents a niche of expert applications that go beyond the capabilities of mainstream manufactured systems. Although the devices we presented may appear technically similar to common wearable sensors [10,88], those used in the performing arts involve specialized implementations designed to meet stringent live performance constraints. These include multi-performer compatibility, long-range wireless transmission, high-resolution data stream, compact form factor [37,45,89,90].

Moreover, our use cases demand both low-latency (typically 10 ms or less) streaming protocols that remain interoperable with computer music systems, such as OSC or MIDI. While most devices today rely on Wi-Fi or BLE (or both), further improvements can be expected as these standards continue to evolve. In particular, the wider availability of technological combinations of IMU with Ultra Wide Band (UWB) could enable cost-effective solutions for absolute device localization in space.

Performing arts impose thus interesting technical requirements on the development of wireless IMUs, requirements that are also relevant to several other application domains. In particular, we are currently developing movement-sonification devices for rehabilitation using the R-IoT devices [4,91]. Notably, rehabilitation with auditory feedback presents requirements very similar to those discussed in this paper, and all the devices described here could be readily adapted for this purpose.

## Figures and Tables

**Table 1:** Features comparison of commercial wireless IMU sensors (to the best of our knowledge on 17 May 2025).

	Sensors	Samplerate (Hz)	Res. (bits)	Protocol	Wireless	Prog.	API
**Mi.Mu**	MARG	>100	unknown	OSC	Wi-Fi	N	N
**MUGIC**	MARG	40–100	16	OSC	Wi-Fi	N	N
**R-IoT 3**	MARG + Baro	200	16	OSC MIDI	Wi-Fi BLE	Y	Y
**SOMI-1**	MARG	200	7–14	MIDI	BLE	N	N
**Wave**	6D IMU	100	7	MIDI	BLE	N	N
**WiDig**	MARG	>100	16	OSC MIDI	Wi-Fi BLE	N	Y
**x-IMU 3**	MARG	400	16	UDP TCP	Wi-Fi BLE	N	Y

**Figure A1:**
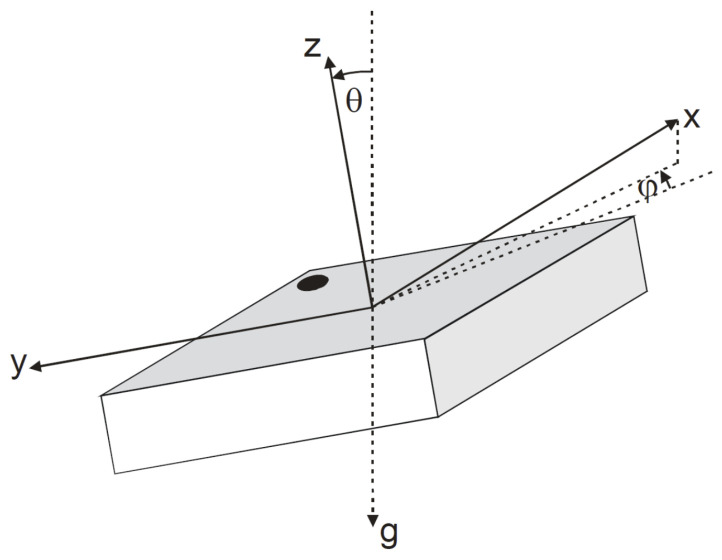
representation of the pitch (θ) and roll (ϕ) referenced to Earth’s gravitational vector.

## Abbreviations

The following abbreviations are used in this manuscript:

ADC	Analog to Digital Converter
API	Application Programming Interface
AM	Amplitude Modulation
ASK	Amplitude Shift Keying
BLE	Bluetooth Low Energy
CPU	Central Processing Unit
CSMA/CA	Carrier Sense Multiple Access with Collision Avoidance
CSMA/CD	Carrier Sense Multiple Access with Collision Detection
DAW	Digital Audio Workstation
DMA	Direct Memory Access
DMI	Digital Musical Instrument
DoF	Degree of Freedom
FM	Frequency Modulation
FSK	Frequency Shift Keying
GATT	Generic Attribute Profile
GRT	Gesture Recognition Toolkit
I2C	Inter-Integrated Circuit
IMU	Inertial Measurement Unit
MARG	Magnetic, Angular Rate and Gravity
MIDI	Musical Instrument Digital Interface
MCU	MicroController Unit
MEMS	Miniature Electro-Mechanical System
ML	Machine Learning
NIME	New Interfaces for Musical Expression
NTP	Network Time Protocol
ODR	Output Data Rate
OSC	Open Sound Control
OSI	Open Systems Interconnection
SPI	Serial Peripheral Interface
SSP	Secure Simple Pairing
TDMA	Time Division Multiple Access
UART	Universal Asynchronous Receiver Transmitter
W3C	World Wide Web Consortium

## Appendix A

accX=1g∗sinθ∗cosϕ

accY=−1g∗sinθ∗sinϕ

accZ=1g∗cosϕ

accY/accX=−tanϕ

leading to:

θ=atan2(accX,accZ)∗180/π

$$\phi =atan2(\left(-accX\right),\sqrt{(accY^{2}+accZ^{2})*180/\pi }$$

Please note that, as already mentioned, not only is the third angle Yaw (heading or compass angle relative to Earth’s magnetic North) missing, but the above computation is only reliable in conditions of stillness. In case of movement, a fusion algorithm using exclusively accelerometer and gyroscope data can be used, such as the complementary filter.
